# Early parathyroid hormone elevation after denosumab therapy following parathyroidectomy: a diagnostic pitfall

**DOI:** 10.1210/jcemcr/luag165

**Published:** 2026-10-16

**Authors:** Hiroyuki Yamashita, Hisakazu Shindo, Daisuke Tatsushima, Yusuke Mori, Kento Katsuyama, Shinya Sato

**Affiliations:** Department of Surgery, Yamashita Thyroid Hospital, Fukuoka 812-0034, Japan; Department of Surgery, Yamashita Thyroid Hospital, Fukuoka 812-0034, Japan; Department of Surgery, Yamashita Thyroid Hospital, Fukuoka 812-0034, Japan; Department of Surgery, Yamashita Thyroid Hospital, Fukuoka 812-0034, Japan; Department of Surgery, Yamashita Thyroid Hospital, Fukuoka 812-0034, Japan; Department of Surgery, Yamashita Thyroid Hospital, Fukuoka 812-0034, Japan

**Keywords:** primary hyperparathyroidism, hypercalcemic crisis, denosumab, postoperative parathyroid hormone elevation

## Abstract

Persistently elevated parathyroid hormone (PTH) after parathyroidectomy is often attributed to residual disease but may reflect altered calcium (Ca) homeostasis. The impact of denosumab on perioperative PTH interpretation remains unclear. A 77-year-old woman presented with a hypercalcemic crisis and was initially managed at another hospital. During treatment with hydration and elcatonin (40 units twice daily), denosumab (60 mg) was administered, resulting in a rapid decline in corrected Ca (c-Ca). Eleven days later, at presentation to our institution, her c-Ca was 9.9 mg/dL (SI: 2.48 mmol/L; reference range, 8.8-10.1 mg/dL [SI: 2.20-2.53 mmol/L]), and intact PTH was 195 pg/mL (SI: 20.7 pmol/L; reference range, 10-65 pg/mL [SI: 1.1-6.9 pmol/L]). At admission, c-Ca decreased further while PTH increased. The patient had chronic kidney disease and vitamin D deficiency. A focused parathyroidectomy was performed for a single adenoma. Thirty minutes after excision, PTH remained above the surgical success threshold and increased further the morning after surgery, while c-Ca levels decreased. Calcium and vitamin D supplementation subsequently normalized both parameters. Denosumab exposure, together with vitamin D deficiency and renal impairment, may cause transient postoperative PTH elevation despite successful parathyroidectomy. Persistent disease should not be assumed when Ca declines despite elevated PTH.

## Introduction

Primary hyperparathyroidism (PHPT) is characterized by the autonomous parathyroid hormone (PTH) secretion, resulting in hypercalcemia and systemic complications. Parathyroidectomy is the only curative treatment. In single-gland disease, cure is typically confirmed by a rapid intraoperative PTH decline followed by serum calcium (Ca) normalization [1, 2]. Therefore, early postoperative PTH reduction is widely considered biochemical evidence of success.

Elevated PTH with normocalcemia may occur after successful surgery [3-6] and does not necessarily indicate persistent or recurrent disease. It is associated with vitamin D deficiency, reduced renal function, and altered Ca–PTH homeostasis [3-6]. An immediate postoperative rise or insufficient PTH decline following adenoma removal is uncommon in patients with single-gland PHPT and may raise concerns regarding incomplete resection or unrecognized multiglandular disease, prompting consideration of re-exploration. The mechanisms underlying these early discordant PTH responses remain unclear.

Denosumab, a monoclonal antibody against nuclear factor κB ligand (RANKL) receptor activator, is used to control severe hypercalcemia [7]. By suppressing osteoclast-mediated bone resorption, denosumab reduces skeletal Ca release and influences systemic Ca–PTH homeostasis [7-9]. However, its potential impact on perioperative Ca and PTH dynamics in patients undergoing parathyroidectomy for PHPT has not been well characterized.

In this case report, we described a PHPT case complicated by hypercalcemic crisis in which denosumab was administered preoperatively. Despite the successful solitary adenoma removal and a postoperative serum Ca decline, intact PTH levels did not adequately decline 30 minutes after excision and increased further on postoperative Day 1, raising concerns regarding persistent disease. Subsequent Ca and PTH level normalization after Ca and vitamin D supplementation suggests a transient physiological response associated with declining serum Ca. To better understand the perioperative biochemical course, additional PTH fraction analyses were performed using stored serum samples. This case highlights the need for cautious interpretation in patients recently treated with denosumab.

## Case presentation

A 77-year-old woman was referred to our institution for surgical treatment of PHPT following initial management of a hypercalcemic crisis at another hospital. Initial laboratory evaluation revealed the following: corrected Ca (c-Ca) was 15.3 mg/dL (SI: 3.83 mmol/L; reference range, 8.8-10.1 mg/dL [SI: 2.20-2.53 mmol/L]), intact PTH was 366 pg/mL (SI: 38.8 pmol/L; reference range, 10-65 pg/mL [SI: 1.1-6.9 pmol/L]), and serum creatinine was 0.84 mg/dL (SI: 74.3 µmol/L; reference range, 0.65-1.07 mg/dL [SI: 57.5-94.6 µmol/L]).

Figure 1 shows the clinical course and changes in serum Ca and PTH levels. Hydration and elcatonin therapy (40 units twice daily) were continued for 16 days, during which denosumab (60 mg) was administered (Day −20). Eleven days after denosumab administration (Day −9), laboratory evaluation showed a c-Ca of 9.9 mg/dL (SI: 2.48 mmol/L) and an intact PTH of 195 pg/mL (SI: 20.7 pmol/L). The patient was admitted 9 days later for definitive surgical treatment (Day −1). On the morning of surgery (Day 0), c-Ca was 9.4 mg/dL (SI: 2.35 mmol/L), while intact PTH was 235 pg/mL (SI: 24.9 pmol/L).

**Figure 1:**
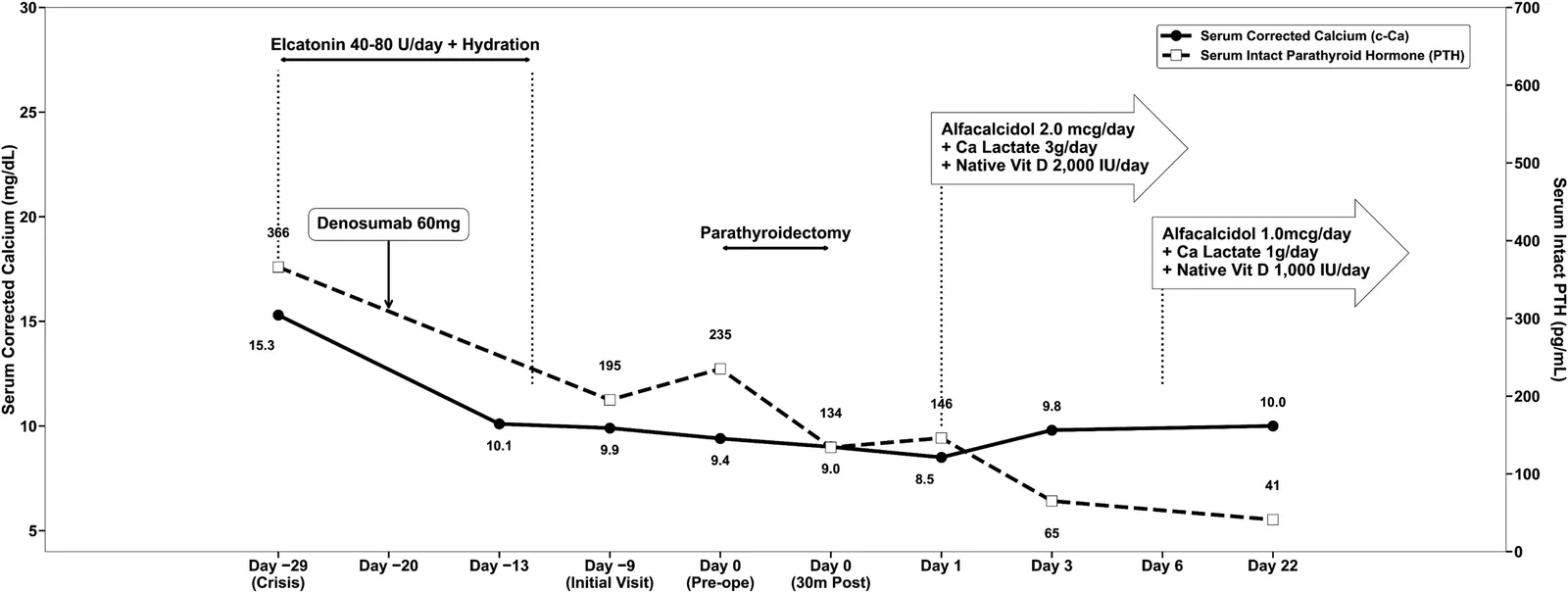
Temporal changes in the corrected calcium and intact parathyroid hormone (PTH) levels from presentation with hypercalcemic crisis through the postoperative period. Denosumab was administered at the referring hospital prior to presentation to our institution. Parathyroidectomy was performed after preoperative localization studies demonstrated a single right inferior adenoma. On postoperative Day 1, corrected calcium declined while PTH increased. Calcium and vitamin D supplementation were subsequently initiated, after which both parameters normalized. Day 0 indicates the day of surgery. Abbreviations: Ca, calcium; PTH, parathyroid hormone.

## Diagnostic assessment

Laboratory evaluation revealed stage G3a chronic kidney disease with an estimated glomerular filtration rate of 59.4 mL/min/1.73 m^2^. Marked vitamin D deficiency was present (25-hydroxyvitamin D, 12.8 ng/mL [SI: 32.0 nmol/L]; reference range, ≥20.0 ng/mL [SI: ≥50.0 nmol/L]). Bone turnover markers showed dissociation: tartrate-resistant acid phosphatase-5b (TRACP-5b) was within the reference range (279 mU/dL [SI: 27.9 U/L]; reference range, 120-420 mU/dL [SI: 12-42 U/L]), whereas bone-specific alkaline phosphatase was found to be slightly above the upper limit (22.9 μg/L [SI: 22.9 µg/L]; reference range, 3.8-22.6 μg/L [SI: 3.8-22.6 µg/L]), consistent with reduced bone resorption in the recent antiresorptive therapy. Furthermore, bone mineral density testing demonstrated osteoporosis (lumbar spine *T*-score, −2.4; femoral neck *T*-score, −3.1).

Preoperative localization studies identified a single right inferior parathyroid lesion on contrast-enhanced computed tomography with concordant focal uptake and persistent delayed retention on ^99^mTc-sestamibi scintigraphy (Fig. 2).

**Figure 2:**
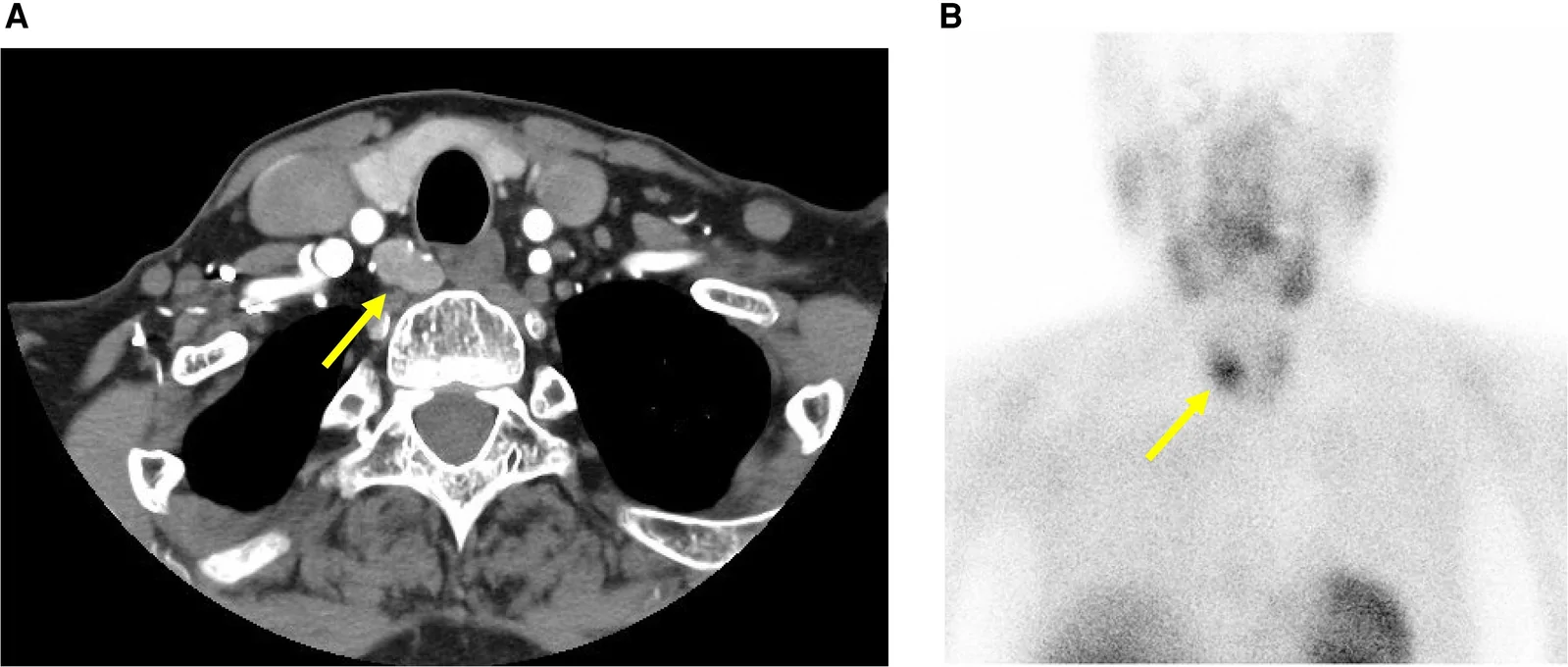
Preoperative localization of the right inferior parathyroid adenoma. (A) Contrast-enhanced computed tomography demonstrating a well-defined lesion posterior to the right inferior thyroid pole (arrow). (B) ^99^mTc-sestamibi scintigraphy showing persistent focal tracer uptake in the right inferior region on delayed imaging (2 hours), consistent with a single parathyroid adenoma (arrow).

To further evaluate the perioperative PTH dynamics, additional biochemical analyses were performed retrospectively (Fig. 3A). Whole PTH (1-84) concentrations were measured using a third-generation assay (Elecsys, Roche Diagnostics). The estimated large C-terminal PTH fragments (eC-PTH fragments) were calculated by subtracting whole PTH (1-84) concentrations from the intact PTH values measured using a second-generation assay [2, 10]. As the C-terminal fragments were not directly measured, these values should be considered approximate estimates. Before surgery, the biochemical course showed declining serum Ca and increasing PTH (Figs. 1 and 3A).

**Figure 3:**
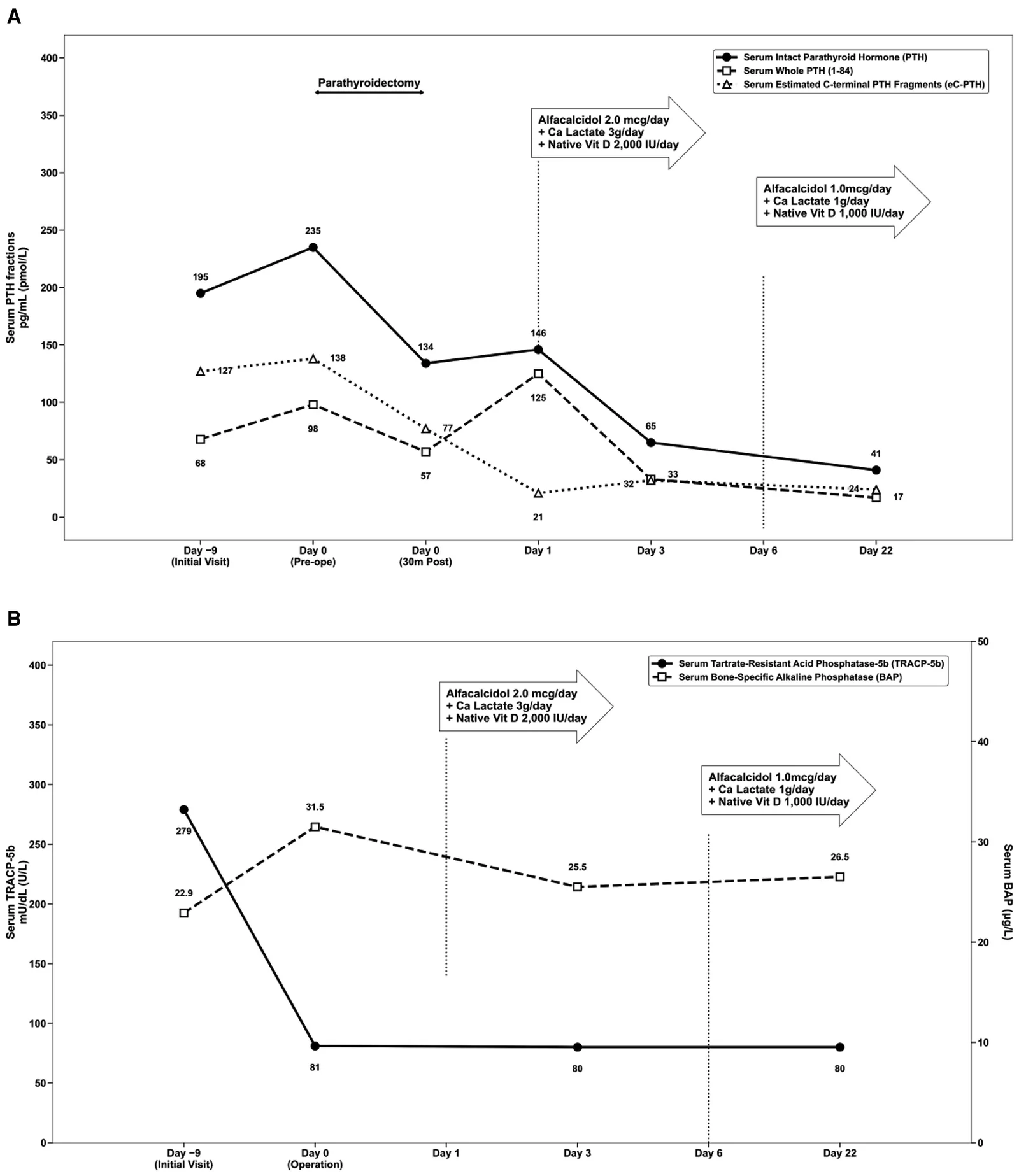
Perioperative changes in the PTH fractions and bone turnover markers. (A) Changes in intact PTH, whole PTH (1-84), and eC-PTH fragments during the perioperative period. Whole PTH (1-84) was measured using a third-generation assay (Elecsys, Roche Diagnostics). eC-PTH fragments were calculated by subtracting whole PTH (1-84) values from intact PTH concentrations measured with a second-generation assay. (B) Changes in the bone turnover markers during the same period, including TRACP-5b and BAP. TRACP-5b was modestly suppressed at presentation (279 mU/dL [SI: 27.9 U/L]) and declined markedly to 81 mU/dL by the time of admission. Although BAP was above the reference range, it was only mildly elevated at the time of surgery and decreased postoperatively. Day 0 indicates the day of surgery. Abbreviations: PTH, parathyroid hormone; eC-PTH, estimated large C-terminal PTH; TRACP-5b, tartrate-resistant acid phosphatase-5b; BAP, bone-specific alkaline phosphatase.

Bone turnover markers were reassessed using stored samples to characterize the skeletal metabolic status during denosumab exposure (Fig. 3B).

## Treatment

Focused parathyroidectomy was performed, and the right inferior parathyroid adenoma was removed (weight, 1722 mg). At our institution, preoperative PHPT localization relies primarily on 4-dimensional computed tomography in combination with ultrasonography or ^99^mTc-sestamibi scintigraphy. When these modalities concordantly indicate single-gland disease, focused parathyroidectomy is performed without routine intraoperative PTH monitoring for surgical decision-making. In such cases, serum PTH measurements on the day of surgery are obtained after the patient returns to the ward, mainly to confirm and evaluate perioperative PTH kinetics.

In the present case, intact PTH measured 30 minutes after adenoma excision decreased from 235 pg/mL (SI: 24.9 pmol/L) to 134 pg/mL (SI: 14.2 pmol/L), which did not meet the institutional criterion for cure (≥50% decrease). However, given the concordant imaging findings and intraoperative identification of a single enlarged gland, the results were interpreted cautiously, rather than as definitive evidence of persistent disease.

On postoperative Day 1, c-Ca was 8.5 mg/dL (SI: 2.13 mmol/L), and PTH was 146 pg/mL (SI: 15.5 pmol/L). Parathyroid hormone levels typically decline by the first postoperative day when single-gland disease is accurately localized. However, in the present case, PTH increased despite decreased serum Ca. Given the association between vitamin D deficiency, mild renal impairment, suppressed bone turnover, and recent denosumab exposure, a compensatory physiological PTH increase was considered. Therefore, oral Ca and active vitamin D were administered on postoperative Day 1 (Fig. 1). Serum creatinine levels remained stable throughout the clinical course (data not shown), thus indicating no significant change in renal function during the perioperative period.

Histopathological examination revealed a well-circumscribed parathyroid adenoma composed predominantly of chief cells without capsular, vascular invasion, or evidence of multiglandular disease.

## Outcome and follow-up

Two days after starting Ca and vitamin D supplementation, c-Ca and intact PTH levels decreased to the reference ranges (Figs. 1 and 3A). The patient remained normocalcemic throughout the follow-up period.

Long-term follow-up at our institution was infeasible for the patient. However, serum c-Ca and PTH normalization after supplementation suggested that early postoperative PTH elevation was likely a transient physiological response rather than evidence of persistent disease.

## Discussion

The findings suggest that denosumab therapy may alter perioperative Ca–PTH homeostasis, leading to early postoperative PTH elevation and potential concerns for persistent PHPT despite successful adenoma removal. In single-gland disease, circulating PTH levels typically decrease promptly after parathyroidectomy [1, 2], but postoperative levels may also reflect systemic Ca balance and recovery of previously suppressed parathyroid tissue.

In our patient, denosumab therapy, vitamin D deficiency, and mild renal impairment may have contributed to the altered Ca homeostasis presurgery. Denosumab potently inhibits osteoclast-mediated bone resorption through RANKL blockade, markedly reducing skeletal Ca efflux [7-9]. Although effective in controlling hypercalcemia, this antiresorptive effect may limit Ca mobilization in response to PTH [7]. Consequently, when Ca levels decline following medical therapy and adenoma removal, compensatory stimulation of the nonadenomatous parathyroid tissue may occur.

In previous studies, vitamin D deficiency and mildly reduced renal function were common but did not prevent postoperative PTH decline after successful parathyroidectomy [11, 12]. Although these factors may have contributed to the biochemical milieu observed in the present case, they are unlikely to fully explain the observed postoperative PTH elevation. Notably, the early rise observed here differs from prior reports, where chronic kidney disease was linked to delayed PTH normalization and vitamin D deficiency to later PTH re-elevation, rather than an immediate increase the morning after surgery. These findings suggest that denosumab was a key modifying factor rather than the sole cause. The marked suppression of bone turnover further supports its role in altering perioperative Ca–PTH dynamics.

The TRACP-5b levels decreased from 279 mU/dL at presentation to 81 mU/dL at admission (Fig. 3B), indicating marked suppression of bone resorption [7-9]. Reduced skeletal Ca mobilization might contribute to declining serum Ca and compensatory PTH secretion.

Parathyroid hormone fraction analysis provided additional information regarding perioperative PTH dynamics (Fig. 3A) [2, 10, 13]. Preoperatively, circulating PTH included a high proportion of large carboxyl-terminal fragments, suggesting fragment-dominant secretion from adenomas [13]. After adenoma removal, whole PTH (1-84) increased together with reduced c-Ca, resulting in whole PTH predominance relative to eC-PTH fragments. Because intact PTH assays detect both bioactive whole PTH (1-84) and large C-terminal fragments, the composition change may influence interpretation of perioperative PTH measurements [2, 10]. Although the source of postoperative PTH secretion cannot be directly confirmed, physiological secretion from residual nonadenomatous parathyroid tissue is the most plausible explanation.

These findings differ from the mechanism proposed in our previous report describing intraoperative PTH spikes associated with the accumulation of C-terminal fragments [10], which explained delayed intraoperative PTH decline. In contrast, the present case demonstrated a relative increase in whole PTH (1-84) rather than fragment accumulation, suggesting a different mechanism.

Although PTH decreases after surgery, 20% to 40% of patients develop normocalcemic PTH re-elevation during follow-up without recurrence [3-6], likely due to the secondary stimulation of suppressed glands from vitamin D deficiency or reduced renal responsiveness to PTH [3-6]. In such cases, PTH re-elevation typically occurs during postoperative recovery rather than immediately after surgery. However, the present case demonstrates an unusually early PTH increase, suggesting a different mechanism. Secondary stimulation was evident before surgery, as declining Ca levels following denosumab administration were accompanied by increased PTH. Thus, autonomous adenomatous secretion may have coexisted with the secondary stimulation of residual glands at the time of surgery, potentially explaining the incomplete intraoperative PTH decline and early postoperative PTH elevation. This pattern is best interpreted as a form of physiological postoperative PTH elevation rather than persistent disease.

This mechanism differs from that of the hungry bone syndrome. In hungry bone syndrome, a high preoperative bone turnover leads to rapid skeletal remineralization after surgery, causing hypocalcemia and an initial reduction in circulating PTH levels [14]. However, in the present case, bone resorption was strongly suppressed, and postoperative hypocalcemia appeared to have resulted from limited skeletal Ca mobilization rather than accelerated skeletal uptake.

This case has implications for hypercalcemic crisis management in patients with PHPT. Although denosumab can effectively reduce serum Ca levels, its potent and prolonged antiresorptive effect may modify perioperative Ca–PTH dynamics [7-9]. Bone resorption suppression may limit skeletal Ca mobilization when serum Ca decreases, enhancing compensatory PTH secretion. In contrast, preoperative bisphosphonate administration has not been consistently associated with prolonged postoperative PTH elevation [15]. This difference may reflect the more rapid onset and prolonged suppression of bone turnover induced by denosumab when compared with bisphosphonates. Therefore, denosumab may exert a more pronounced effect on perioperative Ca–PTH homeostasis, and postoperative biochemical findings in such patients should be interpreted with caution before concluding that persistent disease is present.

In summary, recent denosumab exposure, vitamin D deficiency, and mild renal impairment may contribute to early postoperative PTH elevation despite successful parathyroidectomy. Declined serum Ca accompanied by increased PTH necessitates careful interpretation of perioperative biochemical findings to avoid misclassification of surgical failure.

## Learning points

- Early postoperative PTH elevation after parathyroidectomy does not necessarily indicate persistent PHPT.
- Recent denosumab exposure may alter perioperative Ca–PTH dynamics by suppressing skeletal Ca mobilization.
- Vitamin D deficiency and reduced renal function may contribute to postoperative PTH elevation; they usually do not prevent postoperative PTH decline after successful parathyroidectomy.
- In patients receiving antiresorptive therapy, early postoperative biochemical findings should be interpreted with caution.

## Data Availability

Data sharing is not applicable to this article, as no datasets were generated or analyzed during the current study.

## Abbreviations

c-Ca: corrected calcium
eC-PTH: estimated large C-terminal PTH fragments
PHPT: primary hyperparathyroidism
PTH: parathyroid hormone
TRACP-5b: tartrate-resistant acid phosphatase-5b

## References

[1] Irvin GL 3rd, Dembrow VD, Prudhomme DL. Clinical usefulness of an intraoperative “quick parathyroid hormone” assay. Surgery. 1993;114(6):1019‐1022; discussion 1022–1023. 8256205

[2] Yamashita H, Gao P, Noguchi S, et al Role of cyclase activating parathyroid hormone (1-84 PTH) measurements during parathyroid surgery: potential improvement of intraoperative PTH assay. Ann Surg. 2002;236(1):105‐111. 10.1097/00000658-200207000-00016 PMC1422555 12131092

[3] Nordenström E, Westerdahl J, Isaksson A, Lindblom P, Bergenfelz A. Patients with elevated serum parathyroid hormone levels after parathyroidectomy: showing signs of decreased peripheral parathyroid hormone sensitivity. World J Surg. 2003;27(2):212‐215. 10.1007/s00268-002-6600-5 12616439

[4] Yamashita H, Noguchi S, Moriyama T, et al Reelevation of parathyroid hormone level after parathyroidectomy in patients with primary hyperparathyroidism: importance of decreased renal parathyroid hormone sensitivity. Surgery. 2005;137(4):419‐425. 10.1016/j.surg.2004.12.009 15800489

[5] Lang BHH, Wong IYH, Wong KP, Lo CY. Eucalcemic parathyroid hormone elevation after parathyroidectomy for primary sporadic hyperparathyroidism: risk factors, trend, and outcome. Ann Surg Oncol. 2012;19(2):584‐590. 10.1245/s10434-011-1846-5 PMC3264855 21732144

[6] Lee JE, Hong N, Kim JK, et al Analysis of the cause and management of persistent laboratory abnormalities occurring after the surgical treatment of primary hyperparathyroidism. Ann Surg Treat Res. 2022;103(1):12‐18. 10.4174/astr.2022.103.1.12 PMC9300437 35919112

[7] Thosani S, Hu MI. Denosumab a new agent in the management of hypercalcemia of malignancy. Future Oncol. 2015;11(21):2865‐2871. 10.2217/fon.15.232 PMC4976858 26403973

[8] Bekker PJ, Holloway DL, Rasmussen AS, et al A single-dose placebo-controlled study of AMG 162, a fully human monoclonal antibody to RANKL, in postmenopausal women. J Bone Miner Res. 2004;19(7):1059‐1066. 10.1359/JBMR.040305 15176987

[9] Makras P, Polyzos SA, Papatheodorou A, Kokkoris P, Chatzifotiadis D, Anastasilakis AD. Parathyroid hormone changes following denosumab treatment in postmenopausal osteoporosis. Clin Endocrinol (Oxf). 2013;79(4):499‐503. 10.1111/cen.12188 23452098

[10] Yamashita H, Shindo H, Yoshimoto K, et al Intraoperative parathyroid hormone measurement pitfalls: parathyroid hormone spikes with carboxyl-terminal parathyroid hormone fragments in primary hyperparathyroidism-a case report. Surg Case Rep. 2024;10(1):102. 10.1186/s40792-024-01903-z PMC11045703 38662187

[11] Yamashita H, Noguchi S, Uchino S, et al Vitamin D status in Japanese patients with hyperparathyroidism: seasonal changes and effect on clinical presentation. World J Surg. 2002;26(8):937‐941. 10.1007/s00268-002-6622-z 12016478

[12] Yamashita H, Noguchi S, Uchino S, et al Influence of renal function on clinico-pathological features of primary hyperparathyroidism. Eur J Endocrinol. 2003;148(6):597‐602. 10.1530/eje.0.1480597 12773130

[13] Yamashita H, Gao P, Cantor T, et al Large carboxy-terminal parathyroid hormone (PTH) fragment with a relatively longer half-life than 1-84 PTH is secreted directly from the parathyroid gland in humans. Eur J Endocrinol. 2003;149(4):301‐306. 10.1530/eje.0.1490301 14514344

[14] Paruk IM, Govind K, Pirie FJ, Motala AA. Postoperative hungry bone syndrome in primary hyperparathyroidism: risk factors and outcomes at a South African tertiary centre. BMC Endocr Disord. 2026;26:27. 10.1186/s12902-025-02140-7 PMC12865997 41449412

[15] Etemadi R, Mansouri A, Salehidoost R, Tabatabaei ET. Bisphosphonate therapy and the occurrence of the hungry bone syndrome after surgery for primary hyperparathyroidism. Int J Endocrinol. 2025;2025(1):8285521. 10.1155/ije/8285521 PMC12566956 41163950

